# A Review of the Use of Titanium for Reinforcement of Masonry Structures

**DOI:** 10.3390/ma15134561

**Published:** 2022-06-29

**Authors:** Fitsum Haile, Jill Adkins, Marco Corradi

**Affiliations:** 1 Department of Mechanical and Construction Engineering, Northumbria University, Newcastle upon Tyne NE1 8ST, UK; f.m.haile@northumbria.ac.uk; 2 Perryman Company, Houston, PA 15342, USA; jadkins@perrymanco.com; 3 Department of Engineering, Perugia University, 06125 Perugia, Italy

**Keywords:** titanium alloys, masonry structures, retrofit solutions, earthquake engineering

## Abstract

Titanium has exceptional durability, very high specific strength, a thermal expansion coefficient similar to construction materials, low weight density, and its cost has drastically decreased over the last decades. One of the main requirements in conservation engineering is the durability of the retrofit materials and the reversibility of interventions, and a possible interesting solution is the use of titanium alloys coupled with inorganic matrices made of low-cement or lime mortars. Titanium has recently been used to reinforce important masonry and archeological monuments, but little is known about this. Its use is increasing in conservation engineering without adequate knowledge of its characteristics, grades, and properties. This paper summarizes the main features of titanium alloys, its recent applications, and discusses its drawbacks and advantages compared to other retrofit materials and methods. It is demonstrated that titanium alloys can be effectively used in many applications to reinforce masonry structures while complying with requirements in terms of durability, compatibility, and reversibility. Given its mechanical properties, its use in the repair and reinforcement of masonry structures could be particularly interesting in seismically prone areas.

## 1. Background

Titanium is rarely used in civil engineering, with applications limited to medical implants or aerospace engineering. In conservation engineering, applications are uncommon and not well documented, and this paper aimed to summarize the state-of-the-art research of the use of titanium to reinforce or repair existing masonry structures.

Titanium is typically used under the form of an alloy. The addition of elements, such as molybdenum, vanadium, aluminum, and zirconium, alters the strength, ductility, formability, weldability, and other useful characteristics and properties. Titanium alloys present superior specific strength, high yielding strength (500–1000 MPa), and light weight density (4500 kg/m^3^), which makes it an ideal solution not only in earthquake engineering, where ductility and lightness are fundamental properties for materials [1,2], but also in many other civil applications. Furthermore, titanium can withstand harsh environmental conditions, including salt water and road salts, without demonstrating the usual corrosion problems of carbon steel.

Titanium alloys can be separated into three categories: α (i.e., a hexagonal close-packed structure), α + β, and β (i.e., a centered cubic structure). These categories describe the origin of the crystallographic microstructure. Alloying elements can generally be classified as α or β stabilizers and can alter the microstructure and mechanical properties. Table 1 summarizes the main mechanical properties of different titanium alloys. α Alloys are non-heat treatable and are generally very weldable but exhibit low tensile strength; α + β alloys are heat treatable to varying extents and most are weldable with the risk of some loss of ductility in the weld area. However, the most common and readily available grade of titanium is Ti-6Al-4V (α + β). This is the alloy typically being used in civil engineering applications, with a specific strength (tensile strength/weight density) approximately four times greater compared to S275 carbon steel. It can also be noted that titanium alloys may exhibit elastic deformations (Young’s moduli) and yield strengths significantly larger compared to S275 [3].

**Figure 1:**
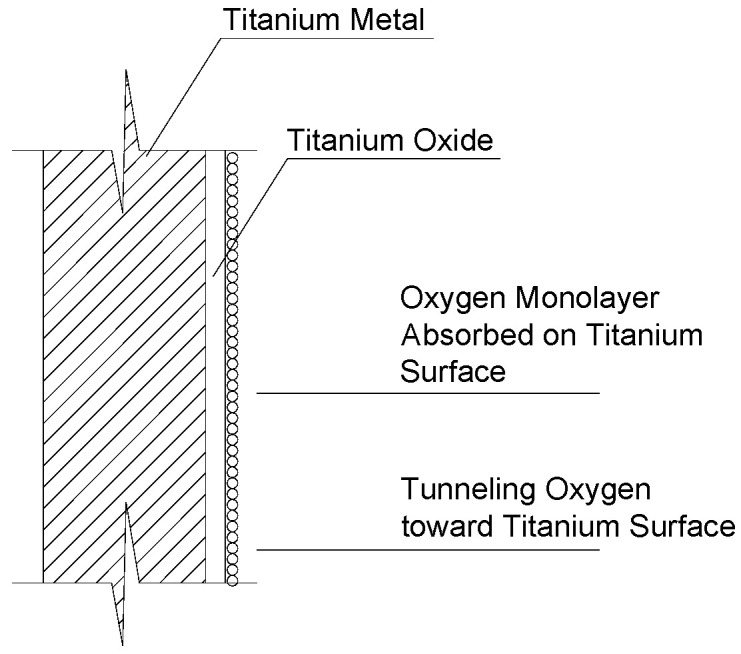
The durability of titanium is facilitated by oxide film formation [4].

However, resistance to corrosion is surely the most important characteristic of titanium. Titanium and its alloys are easily passivated metals. The passivation film (Figure 1) on the surface is very stable and has excellent corrosion resistance. This makes titanium alloys interesting in civil engineering where interventions are often exposed to physical weathering.

**Figure 2:**
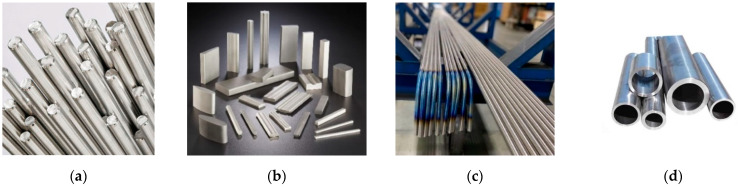
Different titanium elements on the market: (**a**) bars; (**b**) net shapes; (**c**) threaded rods; (**d**) pipes and tubes.

Different titanium shapes are actually present (Figure 2) on the market. The most common types are smooth bars and pipes, but threaded rods and net shapes can also be found. The cost of these structural elements not only depends on the shape but also on their dimensions. Small diameter rods cost more than large diameter ones, because of the higher processing and manufacturing work involved.

Finally, another significant problem associated with the use of titanium in civil engineering is its cost. Most people associate titanium with an expensive technology. Titanium is more expensive than structural steel and stainless steel, but the cost of the material is only a small part of the overall cost of a retrofit intervention. In conservation engineering, the amount of reinforcement material is limited, and the majority of costs are from labor and installation.

## 2. Titanium in Civil Engineering

The use of titanium alloys in civil engineering is rare, but applications are increasing in number. This is the consequence of two important facts: (1) the cost of titanium has reduced drastically over the last decades; (2) recent collapses of civil infrastructures have highlighted the shortcomings of the use of carbon steel in structures exposed to outdoor environments.

**Figure 3:**
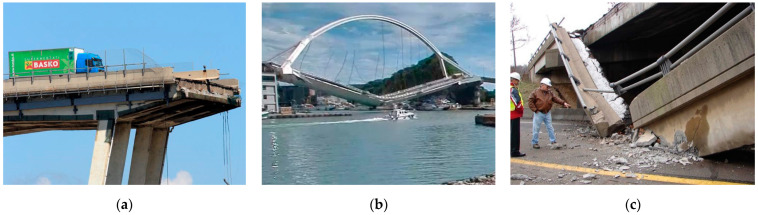
Recent collapses of reinforced concrete bridges due to the corrosion of steel: (**a**) Genoa; (**b**) Taiwan; (**c**) Pennsylvania.

Civil structures and infrastructures have a design life typically much longer than any other aerospace or medical application. In many developed countries, concrete bridges built during the economic boom of the 1950s and 1960s often had inadequate steel reinforcement and need repair or replacement (Figure 3). These bridges are normally still in use after 70 years, and the disadvantages of replacement are high, not only for the economic cost but also in terms of carbon emissions and low sustainability. Recently, titanium was used to rehabilitate and extend the life of bridges saving cost and time over replacement [5,6,7].

A study that began at Oregon State University calculated that “the overall cost for the titanium strengthening for the Mosier concrete bridge was less than 3% of the estimated cost for bridge replacement and 30% lower than rehabilitation completed using alternative materials” [6]. By taking advantage of the unique properties of titanium, such as its high strength and corrosion resistance, it can become the most cost-effective solution when used in the right application.

The situation of old masonry structures or infrastructures is more complicated, not only because of the pre-industrial methods used for their construction but also for their long design life, often longer than 100 years. In Europe, a very large number of masonry buildings and infrastructures were constructed several centuries ago. Governments’ policy is to preserve them, not only for their cultural and social value, but also because their reuse has significant positive aspects in terms of reductions in CO_2_ emissions and sustainability compared to new constructions. In this context, the durability of the reinforcement materials is even more important than other characteristics.

Old masonry buildings, often made of solid bricks or rubble stone masonry, have been typically designed in the past with little or no regard for the effects of seismic loadings. Recent destructive earthquakes in Italy, Greece, Japan, and other areas of the world have clearly demonstrated that these masonry structures are particularly susceptible to the inertial forces activated during earthquakes. The poor performance of old masonry buildings both against the in-plane and out-of-plane horizontal actions, led standardization committees to significantly increase the code requirements for lateral support of existing masonry buildings [8]. With each new earthquake, especially when they produced a significant loss to cultural heritage, reinforcement strategies have been updated and new solutions proposed. However, existing masonry buildings remain at risk because these heritage structures cannot be sufficiently improved to meet the current standards for new masonry constructions [8,9,10].

Masonry constructions have demonstrated a reliable structural performance, but this is basically limited to gravitational static loads [11]. The main problem of the masonry material is its low, almost negligible, tensile strength. Because masonry is bonded into an integral mass by mortar and blocks, it is sometimes considered to be a homogeneous material. However, masonry is clearly a combination of different materials, and this determines the performance of the masonry as a structural element. The mechanical properties of the constituent materials and the interaction of the materials as an assemblage have a significant effect on its behavior. While stones, especially when they are hard, and bricks typically exhibit satisfactory/good mechanical properties, both in compression and in tension, the mortar in old constructions is always made with limes, and its compressive strength typically ranges between 1 and 5 MPa, but its tensile and bond strengths are rarely greater than 0.1–0.15 MPa [12]. Furthermore, mortar-to-block bonding is often very weak, especially when the assemblage is subject to shear or tensile stresses; its strength is even smaller than the mortar tensile strength. In historic buildings, it is common to find tensile cracks as a result of low-intensity seismic events and land subsidence and settlings due to the seasonal changes in moisture contents and temperature profiles within the foundations. While it is well accepted that these cracking phenomena are not very serious, the problem becomes much more serious if the buildings are located in areas exposed to seismic or flooding hazards [13].

In the late 1990s, the use of composite materials (i.e., FRP, fiber-reinforced polymers) was proposed as a viable retrofitting method for heritage masonry structures. A large amount of research has been conducted to assess the structural response of masonry structures reinforced with epoxy-bonded FRPs. While it has been demonstrated that composite materials are able to increase the seismic capacity of masonry members, recent research has also highlighted the poor durability of these retrofits. Phenomena of mechanical degradation, debonding from masonry, and cracking deterioration were observed on FRP-reinforced masonry after only few years after application [14,15].

Another significant shortcoming of FRPs is their low shear and tensile strengths (along the directions perpendicular to the composite fibers). In general, FRPs exhibit a very high tensile strength along the direction of the fibers, but it is not easy in conservation engineering to apply the fibers in such a way that loads on the reinforced masonry structure will only activate the tensile strength of the composite reinforcement. Low-magnitude shear loads may produce premature cracking in the composite, especially when used under the form of FRP strips and sheets.

The use of an isotropic material, such as steel or titanium, can solve this problem. The shear strength of these metals is surely higher than any other composite material, and this can simplify the design load, also covering uncertainties in terms of the directions of the load and second-order effects.

However, this passivation film (Figure 1) may highly reduce bonding of titanium elements with other materials. The problem needs to be better addressed; an earlier study by Osofero et al. [16] demonstrated that titanium alloy tubes exhibit significantly lower bonding strength to mortar compared to carbon steel.

### 2.1. Reversibility and Multidisciplinarity in Conservation

While it is evident that reinforcement of masonry heritage structures and their protection from the effects of natural and manmade hazards (earthquakes, flooding, impacts, fire, etc.) are the mission of a structural engineer, the conservation of these structures involves many other competences. This is a peculiar situation, very different from other engineering areas where structural and cost considerations prevail in the design process. The points of view of conservators, architects, historians, and policymakers may govern the decision-making process. The concept of “heritage value” and the risk of heritage loss and its impact on societies are to be considered when intervening on a monument, and a multidisciplinary approach is necessary. This clearly may have an effect on the type of intervention and the choice of retrofit materials, sometimes overshadowing its economic cost.

It should be mentioned here the International Council on Monuments and Sites (ICOMOS) guidelines [17] for interventions on architectural heritage structures, requiring that, “Where possible, any measures adopted should be “reversible” so that they can be removed and replaced with more suitable measures when new knowledge is acquired. Where they are not completely reversible, interventions should not limit further interventions”. This can have a significant effect on the design of interventions: epoxy-bonded reinforcements should be avoided, since these are difficult to be removed (nonreversible). Conservation bodies do not often authorize the use of materials, such as unprotected carbon steel and FRPs, for the monuments in their portfolio. Reinforced-concrete retrofits are also not recommended and often prohibited because of the low compatibility of cement-based products [18].

**Figure 4:**
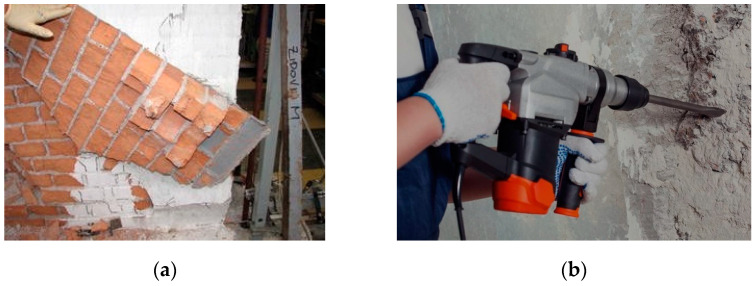
Example of nonreversible retrofit interventions: (**a**) epoxy-bonded FRP reinforcement is very difficult to be removed, and this causes damage to the masonry material; (**b**) reinforced concrete jacketing can only be removed with a demolition hammer.

Figure 4 shows examples of two nonreversible retrofit interventions: epoxy-bonded FRP and Reinforced Concrete jacketing. The use of epoxy-bonded composites to reinforce shear walls is nonreversible, because the epoxy penetrates into the masonry, and the phenomenon of “masonry peeling” occurs when someone tries to remove it. Reinforced concrete jacketing involves the use of noncompatible materials (cement), which is also difficult to be removed without damaging the masonry material.

Although only a very small portion of heritage masonry buildings and infrastructures are listed by ICOMOS (UNESCO list), the approach used by national and local conservation bodies is similar: reversibility and compatibility are key aspects in conservation engineering. These bodies oversee tens of thousands of buildings. The number of listed buildings has increased dramatically over the last decades in Europe as a consequence of the larger interest in architectural heritage, local history, social traditions, and the development of cultural tourism. In some countries, such as Italy, public buildings are automatically listed after 70 years from their construction.

### 2.2. Titanium Corrosion Resistance

In conservation engineering, the most interesting use of titanium is under the form of bars to be inserted in critical weak regions of a masonry building to improve the building’s structural response. These bars are typically coupled with lime mortars, and the strengthening effects fully depend on the bonding behavior of the titanium to mortar. Despite its importance in the performance of the retrofit intervention, the bond between titanium and mortars has not been fully investigated. An interesting solution could be the use of ribs, similarly to rebar in concrete, or other forms of superficial treatments (threading, knurling, chemical treatment, etc.). The mortar between consecutive ribs can facilitate a mechanical interlock between both materials that end up anchoring the titanium rods, restraining the relative displacement. However, ribbed and threaded titanium rods are difficult to find on the market, and more research is needed to investigate the weakening effect of the oxide film on the mortar-to-titanium bond [19].

The “history” of new materials in conservation engineering had a decisive push after the 1997 Central Italy earthquake: the collapse of the masonry vaults with the famous Giotto’s and Cimabue’s frescos at Assisi’s Basilica in Italy clearly demonstrated the limitations of traditional reinforcement and repair methods used so far in historic constructions [20]. The timber roof of the Assisi’s Basilica had been previously replaced with a reinforced concrete structure; the increase in weight and the low deformation capacity of the new concrete roof had major roles in the collapse of the basilica.

The scientific community began to look at alternative “light” retrofitting solutions and composite materials, mainly made of carbon and glass fibers, sparking the interest of structural engineers, as a very high tensile strength and low weight are the main characteristics of FRP. However, it took several years to understand the important shortcomings of FRPs: low reversibility, chemical hazards for workers, low durability, low resistance to high temperatures, and low strength perpendicular to the direction of the fibers.

In this situation, practitioners and researchers reverted to more traditional metal reinforcements, also considering their positive ductile behavior, ease of application, reversibility, and limited cost. In order to improve the effectiveness of the metal reinforcements, new materials and methods have been experimented. An example is the use of ultrahigh strength steel fibers [21,22] and stainless-steel profiles [23,24]. Composite materials continued to be used for historic masonry reinforcement, but their application was made not with epoxy adhesives but with metal fasteners or inorganic (lime- or cement-based) mortars [25,26,27].

However, the corrosion problems of steel reinforcements are difficult to solve. Corrosion not only reduces the resisting steel sections (and this could compromise the reinforcement action), but rusting can seriously damage historic masonry members and cultural heritage assets. For these reasons, conservation bodies also limit the use of steel reinforcement on listed buildings. Creep is another critical aspect to consider when steel reinforcements are applied.

Corrosion-resistant materials have been investigated, and aluminum reinforcements could represent an interesting alternative, but the low modulus and low tensile strength of aluminum alloys can be problematic in many applications.

The use of stainless steels and titanium alloys was soon considered as viable. Both materials are not new: stainless steel has been employed in the military industry since late 19th century, and titanium alloys have been used in aerospace construction since the 1960s.

The main alternatives to the use of titanium are stainless steels and composite materials. New types of stainless steels with high pitting resistance equivalent (PRE) numbers entered the market in the 2010s. The PRE number depends on the steel’s chemical composition (PRE = Cr(%) + 3.3(Mo)) and provides an important indication of its corrosion resistance.

**Figure 5:**
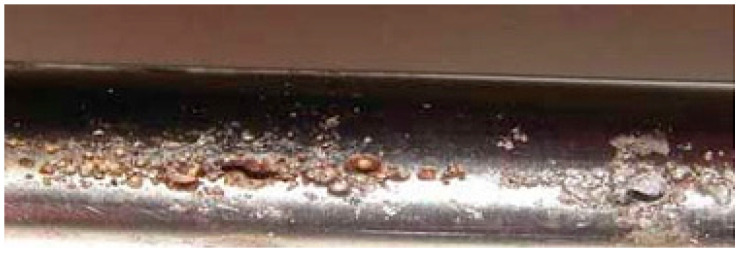
Pitting corrosion on stainless steel [28].

A large number of interventions and technical solutions have recently been proposed using stainless steels: confinement by wrapping of masonry columns, shear reinforcement of wall panels, out-plane-plane reinforcement of wall facades, etc. However, there is general misinformation regarding the corrosion resistance of stainless steel: a very large number of austenitic stainless steels (basic grades 304 and 316) exhibit relatively low corrosion strengths. Figure 5 shows the common pitting phenomenon of an austenitic steel. New stainless steels, namely Duplex steels, exhibit high PRE numbers. The cost of Duplex steel is, however, much higher compared to standard stainless steels and subject to market variations, especially its components (nickel, chromium, and molybdenum). The cost of high-PRE number nickel alloy 625 (PRE = 52) in November 2021 13 EUR/kg, while Ti-6Al-4V alloy costed approximately 31 EUR/kg. However, the yielding strength of stainless steels is 30–40% lower compared to a titanium alloy, and a much smaller quantity of titanium material is needed in retrofit applications, partially reducing the gap between the cost of the two types of materials.

The PRE number of Duplex steels ranges between 24 and 55. However, several studies have highlighted that stainless steels can be subjected to corrosion phenomena, depending on the environmental conditions and their duration. Recent comparative research of the phenomenon of the corrosion of orthodontic appliances have demonstrated that titanium alloys exhibit a corrosion current density up to 50% smaller compared to stainless steels [29,30].

## 3. Review of the Use of Titanium in Conservation Engineering

Interventions on heritage masonry structures using titanium reinforcements are not common, but their numbers are increasing and escalating. The first applications were made in the 2000s on iconic archaeological monuments. Table 2 summarizes the possible applications where titanium alloys could be or have been used in real applications. This table also provides an analysis of the benefits/cost ratio; the outcome of this analysis was typically positive when the amount of titanium needed is low and exposed to unprotected environments. Another factor governing this analysis was the cultural and social value of the monument; clearly for iconic, high-cultural value monuments, the importance of the monument justifies the use of an expensive material, such as titanium, given its features in terms of mechanical properties, durability, and deformation capacity.

**Figure 6:**
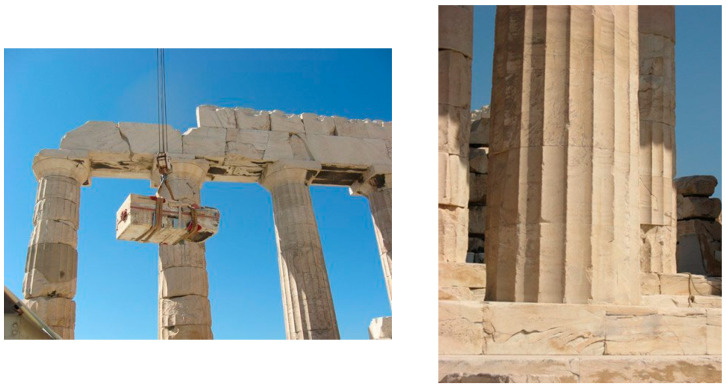
Intervention at the Parthenon, Athens, Greece.

**Figure 7:**
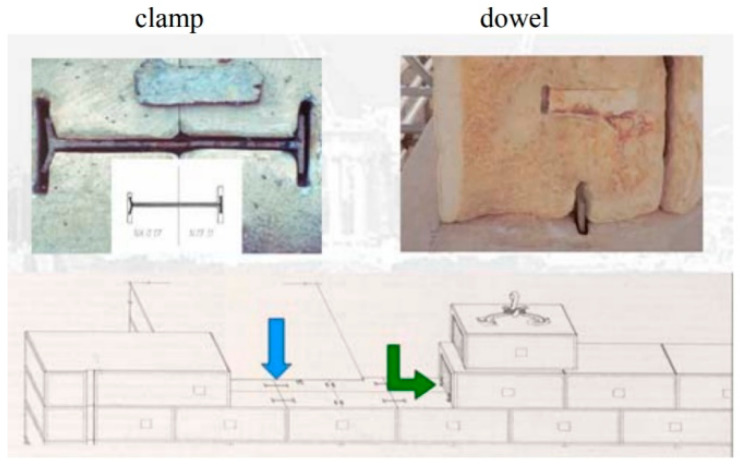
Details of the connection method used to join stone blocks using titanium ties and a special cement-based mortar at the Parthenon [31,32].

An interesting intervention using titanium clamps and dowels was carried out in 2008–2010 at the Parthenon temple in Athens, Greece (Figure 6) [31]. The main structural problem was to efficiently connect large squared megalithic hard stones (made of marble), used in the lateral walls and in the columns of the temple to prevent the sliding phenomenon and to improve the seismic capacity of the construction (Figure 7). The use of titanium allowed for adopting an approach of “trying to restore the maximum amount of ancient masonry while applying the minimum amount of new material. That means using clamps and rods made of titanium—which won’t corrode and crack the marble—and soluble white cement, so that repairs can be easily undone should future generations of restorers discover a better way” (comment by the director of the Acropolis Restoration Project, M. Ioannidou) [31,32].

First attempts previously conducted using carbon and stainless steel caused stone cracking due to the very different thermal expansion coefficients of the stone and stainless-steel elements. Titanium alloys typically exhibit a linear thermal expansion coefficient (approximately 8.5–9 10^−6^ °C^−1^) 25–30% smaller compared to stainless steel. The replacement of steel with a titanium alloy has solved this problem, the high tensile strength of titanium minimized the amount of new material, and its high corrosion resistance was sufficient also for interventions exposed to severe weathering and intended to last for decades.

**Figure 8:**
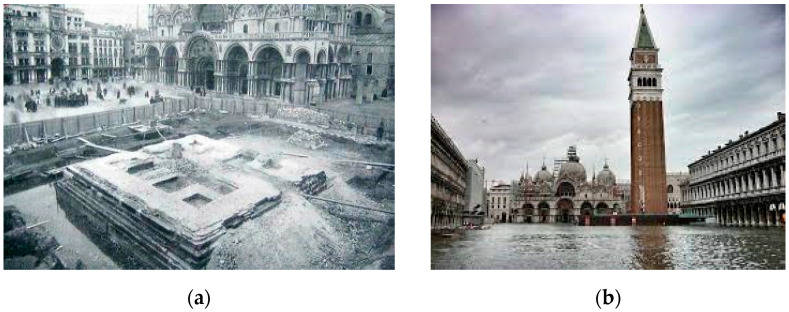
(**a**) Reconstruction of the St. Mark bell tower, Venice, Italy, in the 1910s, made of bricks and stones over timber piles; (**b**) the bell tower, after reconstruction, with the flooded square and foundations. High tide often causes flooding in St. Mark square.

**Figure 9:**
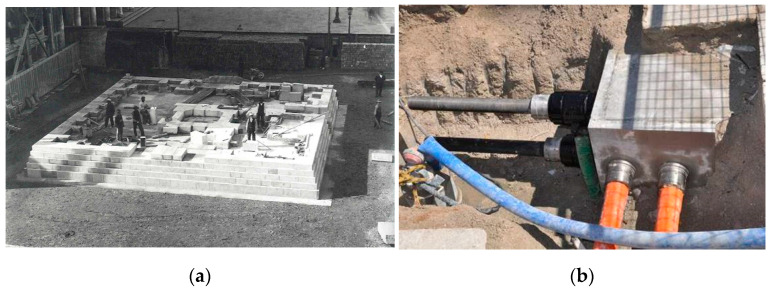
(**a**) Schematic of the bell tower, Venice, Italy; (**b**) titanium rods installed to confine the brickwork and timber piles at the foundations level (design and calculations G. Macchi and G. Galeazzo).

Titanium ties have been more recently used to stabilize, at the base, the bell tower of the St. Mark cathedral in Venice (Figure 8 and Figure 9) [33]. The bell tower was constructed on wooden piles sunk into the lagoon and has suffered from gradual subsidence, which caused the massive construction (98.6 m in height) to slowly sink and slide downwards into the polluted water. Titanium was found to be an ideal application, given the very aggressive environment due to the continuous flooding with salt water and its filtration into the bell tower’s foundations from the nearby sea. The retrofitting solution consisted of wrapping the wooden piles to prevent lateral buckling and the overlaying brickwork walls using titanium ties.

**Figure 10:**
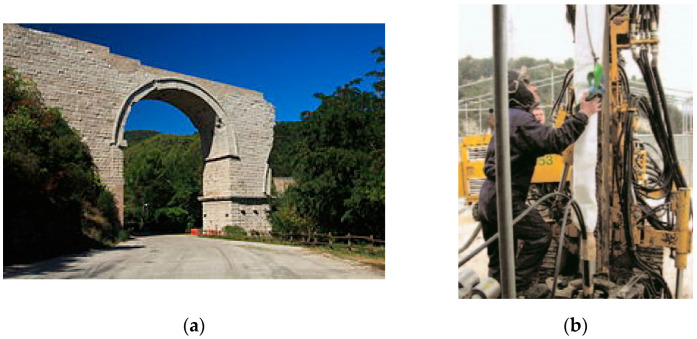
(**a**) Reinforcement of a Roman bridge in Narni, Italy, with titanium rods. The intervention was aimed at containing the effects of the arch thrust. (**b**) Titanium ties were installed vertically in the pier (into the Roman concrete) (design and calculations L. Marchetti and P. Angeletti) [2,34].

Figure 10 shows another recent application. Titanium bars were inserted into the “concrete” material of a Roman bridge in Narni, Italy, to absorb the arch thrust. In Roman times, perfectly cut hard stones were used in the construction of the external perimeter of the walls, while the inner core was filled with a concrete made of fine aggregates and lime. Since a portion of the bridge collapsed in the 11th century, the concrete part remained exposed to environmental actions and was under the effect of the thrust of the arch. A significant crack pattern was noted both in the concrete and masonry materials. In 2007, the engineer opted to repair the concrete material, using compatible limemortars, able to resist the leaching phenomena and to install titanium rods. In doing so, it was possible to stabilize the pier (Figure 10b). The intervention consisted of drilling vertical cores (diameters of approximately 24 mm) and inserting the titanium rods. The titanium rods, 45 m in length, were anchored in the underlaying bedrock up to a depth of 7 m. Rods were installed in pieces of 3 m each and assembled onsite using cylindrical connector nuts. In order to promote reversiveness, no grout was used to fill the holes where rods were inserted. A steel plate and a nut were applied at the top, and a torque wrench was used to compress the masonry/concrete and prestress the titanium rods [2,34].

**Figure 11:**
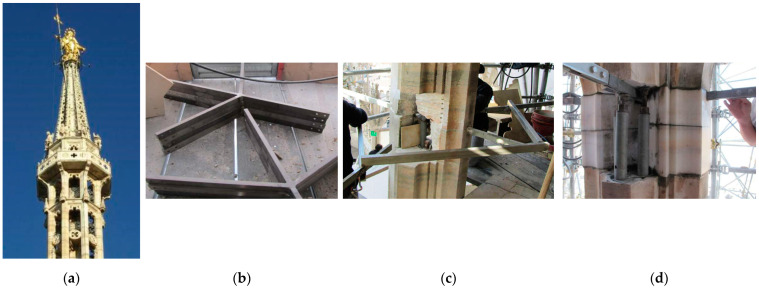
Reinforcement of the main spire of the Milan Cathedral (**a**); details of the titanium anchor plate (**b**); installation method (**c**); titanium rod tie (**d**).

Another structural application of titanium solid-rod elements was carried out in 2010–2012 on the main spire of the Milan Cathedral [35]. This arched vertical cantilever beam is more than 17 m high (Figure 11a). The horizontal thrust of the arched structure was contrasted by means of titanium alloy ties inserted in the marble piers of the spire. The work consisted of the temporary removal of the marble arch segments to install the titanium anchor plate (Figure 11b–d). Unfortunately, the limited literature available on this and previous applications of titanium elements does not specify the dimensions of the titanium profiles, titanium grade, and construction details.

**Figure 12:**
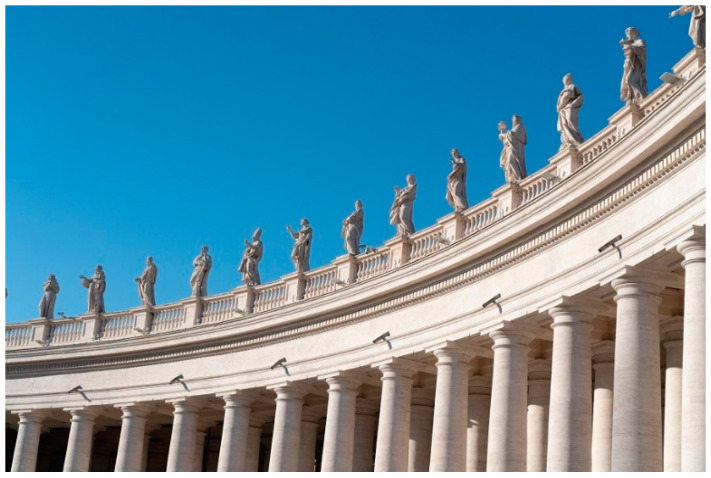
17th century Bernini statues and colonnades, Vatican City. The marble columns are 16 m in height, and the overlying statues are 3.1 m. Cracking was recently detected in several statues and columns, and titanium reinforcements were used for repair.

**Figure 13:**
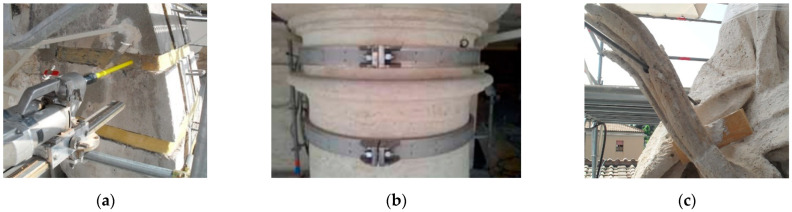
Bernini’s statues and colonnades, Vatican City: (**a**) transversal drilling to restore continuity of the cracked marble using titanium rods; (**b**) wrapping of marble columns; (**c**) projecting parts of the statues and columns were retrofitted using titanium rods [36].

More recently, consolidation interventions have been carried out on iconic monuments in Rome. Here, we can mention the Bernini’s statues and colonnades in St. Peter’s square, Vatican City. The colonnade (Figure 12) consists of 284 marble columns having a height of 16 m. The columns were installed in 1655 and were transported by river on the Tiber from the nearby city of Tivoli. Bernini also carved 140 marble statues (3.1 m height) of saints. Several vertical cracks were noted in the columns due to the high compressive stresses, while some extremities of the marble statues tended to detach. The intervention consisted of wrapping the columns with titanium elements and aramidic fibers in the form of unidirectional sheets. The continuity in the marble material was restored using FRP and titanium rods, inserted in transversal perforations (Figure 13).

**Figure 14:**
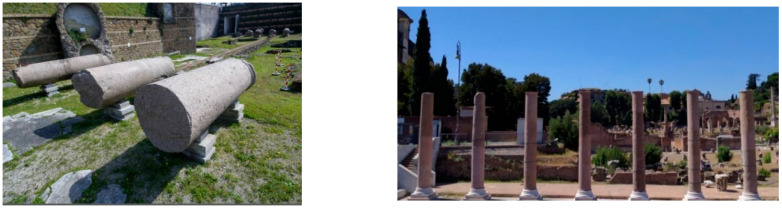
Temple of Peace, Rome. The marble columns were retrofitted with titanium wraps and seismically isolated at the base with dampers [37].

A recent intervention was carried out in Rome at the Temple of Peace [37]. The discovery of a large number of cylindrical marble blocks, in a horizontal position and constituting the portico leading to the entrance of a building, induced the Italian Ministry of Culture to re-assemble part of the temple. The main structural problem was the crack pattern (vertical cracks in the marble blocks), the block-to-block connection and the seismic capacity of the reconstructed structure. Seven meter high marble columns were re-assembled, placed vertically, and reinforced using titanium alloy wraps (Figure 14). These titanium elements were installed with low pre-tension over the cracked area in order to activate a confinement effect. The columns were also seismically isolated using a damper connected with a steel tie placed vertically by coring the marble columns.

## 4. Applications under Study

**Figure 15:**
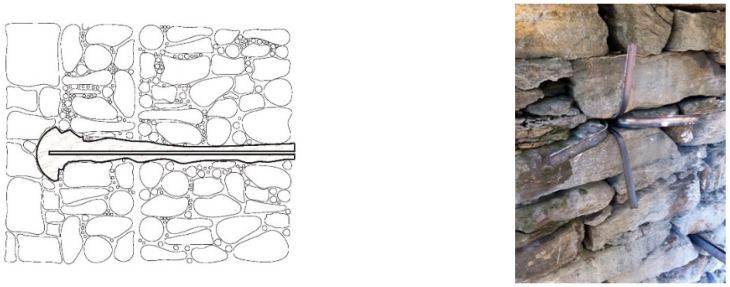
Examples of leaf-to-leaf connections; connections in historic masonry constructions are an area of concern. Metal ties can be inserted using lime mortar.

**Figure 16:**
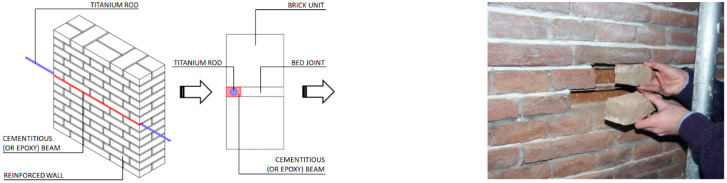
Shear reinforcement of wall panels: the tensile strength of historic masonry is typically very low. Under the action of an earthquake, shear cracking is a common failure mode. Metal rods can be embedded into the mortar joint to absorb tensile stresses under seismic loading. Rods are hidden from view, and this can be important when the fair face aspect of the masonry needs to be preserved.

**Figure 17:**
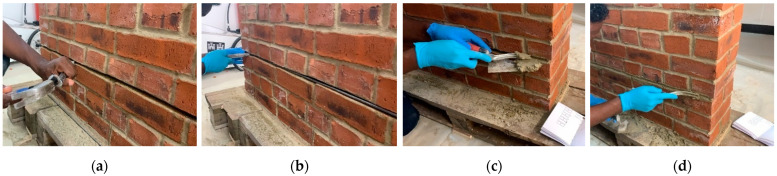
Application phases of titanium alloy threaded rods (bed joint reinforcement): (**a**) removal of the existing mortar from the bed joint; (**b**) application of the titanium rod; (**c**,**d**) repointing with new mortar [43].

**Figure 18:**
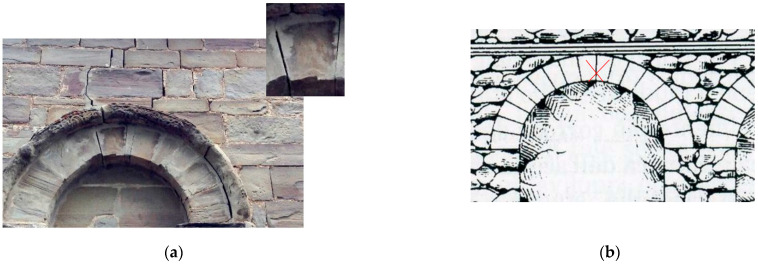
(**a**) Shear loads and differential subsidence can cause ring stones to slide on another, (**b**) Titanium rods, inserted in holes drilled in adjacent blocks, could be used to prevent sliding.

Titanium reinforcements can be used in all applications where metals, such as iron and stainless steels, have been traditionally employed in conservation engineering, with the advantage that the mechanical properties (strength, Young’s modulus, thermal expansion, etc.) and durability of titanium are able to solve a variety of drawbacks common in steels and open the way to outdoor or unprotected applications. Figure 15, Figure 16, Figure 17 and Figure 18 show different solutions under study. For all of them, a very small amount of metal is necessary (typically, the diameter of the metal rod used in these applications is 5–6 mm), making the issue of material cost less important also considering the higher installation and labor costs.

Figure 15 shows a traditional intervention in earthquake engineering: the connection of multi-leaf walls with metal ties. Multi-leaf walls are common in historic construction, and their collapse is frequent under seismic action. There are several methods available on the market to connect wall leaves, and titanium bars could surely spark the interest of structural engineers.

Figure 16 illustrates another well-known application: the reinforcement of bed joints with metal rods. The use of lime compatible mortars to repoint the bed joints exposes the rod to environmental agents with high risks of corrosion. Titanium rods could be an interesting solution, given their high durability. Figure 17 shows the reinforcement phases of an experimental work carried out in 2022 at Northumbria University. Bed joint reinforcement was used to increase the lateral load capacity, and the test results demonstrated that it was possible to increase the lateral capacity of shear wall up to 85% compared to unreinforced brickwork walls [43].

Finally, another interesting application is shown in Figure 18: cracking in historic walls often causes the phenomenon of relative sliding in arches’ stone blocks (Figure 18a). This is a serious structural problem, as it can potentially lead to the collapse of the building. Structural engineers need to “stop” the sliding phenomenon, as a priority, and eventually relocate the stone block to its original position for aesthetic reasons. Titanium bar reinforced perforations, applied alone (“dry” application) or with mortars, could be an ideal solution, given both the high tensile and shear strengths of titanium (Figure 18b).

## 5. Perspectives

**Figure 19:**
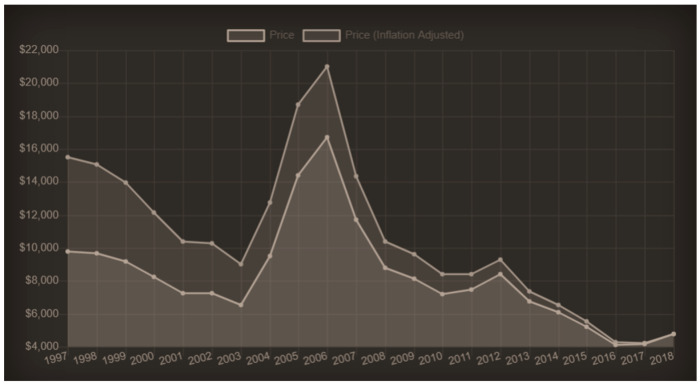
Inflation adjusted cost of titanium: as of January 2016, the price was USD 3750 per ton; in 2005, the price was USD 21,000 a ton [44].

The high cost of titanium is partially due to the high energy consumption needed for fabrication. However, the cost of titanium and its alloys have reduced dramatically over the last decades. From its peak in 2005, the cost has decreased by approximately 80%, and compared to its average cost (inflation adjusted) in the 1990s, the cost of titanium has reduced by 70% (Figure 19). This trend will likely continue in the near future. The problem of corrosion of civil structures and the recent collapse of numerous reinforced-concrete bridges have emphasized the need to use more durable metals in civil applications. Although new grades of stainless steels available on the market may exhibit very high durability (for example Duplex stainless steels), their cost is high and sometimes comparable to that of titanium alloys.

The linear thermal expansion coefficient of many titanium alloys is similar to stone and masonry materials. This is another advantage of titanium compared to steel for applications in conservation engineering. The tensile strength of several titanium alloys is exceptionally high, and this is interesting for applications where stress concentration may occur (restoring the continuity of projecting parts of marble statues, tying of stone blocks, etc.). Surely, the use of titanium in civil engineering will increase in the future, confirming a trend already in place; however, much work remains to be done before titanium alloys may find widespread use within a variety of large-scale civil applications.

## 6. Conclusions

This paper presented an overview of recent titanium applications in conservation engineering, and a discussion of the drawbacks and advantages of titanium compared to other retrofitting materials was introduced.

The main strength of titanium is surely its durability, higher than any other stainless steel or composite material. Interventions on heritage masonry buildings, or existing masonry constructions in general, are typically designed to last for decades, and sometimes for centuries. These buildings are not only exposed to atmospheric agents (high temperatures, frost, solar radiation, humidity, acid rain, etc.) but also to the increasing effects of climate change, resulting in new hazards from natural disasters.

These effects have been partially underestimated not only by professionals and industry but also by the scientific community working in conservation engineering. A large number of interventions using FRPs or stainless steel, carried out in the 1990s and 2000s, show signs of mechanical degradation, debonding of reinforcement from masonry substrate, and corrosion. In some cases, these effects have caused irreparable damage to the masonry structure.

From this review paper, the following conclusions can be drawn:

1. Titanium reinforcement has been successfully used to reinforce archaeological monuments and artefacts. A large number of interventions have been recorded in this area, most of them to restore the continuity of projecting parts of marble statues;
2. Applications of titanium alloys in conservation engineering have only just begun. It has been noted that despite the high cost of titanium, interventions using titanium members are not exaggeratedly expensive, as the low quantity of material required in these interventions and the low cost of installation, compared, for example, to composite materials, where health risks to worker can be significant, make these interventions interesting;
3. Interventions on masonry structures are limited in number. These are often designed to increase the axial load capacity of masonry or marble columns by the confinement effect with titanium alloy wraps. Several interventions have been noted in this category: titanium wraps can be left unprotected end exposed to weathering agents, given the high durability of titanium;
4. The durability of titanium is activated by the passivation film on its surface. However, this film can reduce the bonding with mortar. In retrofitting interventions where titanium is coupled with mortars (for example, bed joint reinforcement or multi-leaf walls tying) this could compromise the retrofitting effect.

It can be concluded that titanium could be an interesting alternative to traditional retrofitting materials, but more research is needed before applications can be designed. Unfortunately, most applications have been conducted so far without adequate experimental validation.

The advantages of the use of titanium in conservation engineering are numerous: it is not only durable, but its deformation properties, both under external and thermal loadings, are similar to that of masonry, highly reducing the risks of producing damage to the masonry because of incompatibility of deformations. In addition, interventions using titanium alloys can be highly reversible.

The analysis of the mortar-to-rod bonding properties appears to be the next logical move in order to facilitate the use of titanium alloys in conservation engineering. However, significant time and effort is required to properly assess the effectiveness of titanium retrofits in the most common structural weaknesses of masonry buildings.

## Figures and Tables

**Table 1:** Mechanical properties of the main titanium alloys.

Material, Category	Young’s Modulus (GPa)	Yield Strength (MPa)	Tensile Strength (MPa)	Elongation at Failure (%)	Weight Density (kg/m^3^)
Ti Grade 1, α	102	138	240	24	4500
Ti Grade 2, α	102	275	345	20	4500
Ti Grade 3, α	102	380	450	18	4500
Ti Grade 4, α	104	483	550	15	4500
Ti-6Al-4V ELI, α + β	95–105	759	828	10	4400
Ti-6Al-4V, α + β	95–105	828	895	10	4400
Steel S275	206	275	370–450	20	7800

**Table 2:** Main applications of titanium in conservation engineering.

Type of Intervention	If Intervention Has Been Made on a Real Monument	Benefits/Cost
Connection of stone block in archeological monuments [31,32]	Yes (approximately 2–10 applications)	High *
Use of titanium solid rod ties in aggressive (sea water) or unprotected environments [2,33,34,35]	Yes (approximately 2–10 applications)	Medium *
Wrapping of marble, brickwork, or stone columns [36,37,38]	Yes (approximately 3–10 applications)	High *
Restoring continuity of projecting parts of marble statues [36,39,40,41,42]	Yes (20–100 applications)	High *
Connection of stone arch segment to prevent sliding	No	High *
Wall-to-wall connection to prevent out-of-plane rocking during earthquakes	No	Medium *
Tying multi-leaf walls (transversal connection of the wall leaves)	No	Medium *
Bed joint repointing of shear walls (in-plane reinforcement) [43]	No	Low *

* The assessment of the benefit/cost ratio highly depended on the cultural and social value of the building or monument to repair or reinforce.
